# Supplementary material for: Are carbon emissions trading and green financial instruments synergistic? -Comprehensive quantitative research based on content analysis
Source: PLoS One. 2024 Mar 7;19(3):e0298601. doi: 10.1371/journal.pone.0298601 (PMC10919601; doi:10.1371/journal.pone.0298601)
Supplement: S1 Data — (ZIP) [file pone.0298601.s001.zip › Data/地级市Z_F工作报告.zip!/╝¬┴╓/═¿╗»╩╨/└┤╫╘╣1⁄2╓┌║┼í╛┬φ┐╦╩2╛▌═°í┐_2012.docx]

一、2011年工作回顾

　　2011年，在省委、省政府和市委的领导下，市政府和全市人民一道，深入实施“三化”统筹、“三动”战略和富民工程，集中精力抓项目、兴产业、惠民生、促发展，圆满完成了市六届人大四次会议确定的各项任务，实现了“十二五”良好开局。

　　经济增长速度和质量大幅提升。预计：全市地区生产总值完成760亿元，比上年增长21.2%，增幅为近三年最高；规模以上工业总产值跃上千亿元台阶，达到1200亿元，增长43.5%；一般预算全口径财政收入完成80.2亿元，地方级财政收入完成45.1亿元，分别增长30%、32.5%；固定资产投资完成600亿元，增长36.7%；社会消费品零售总额完成290亿元，增长17.4%；节能减排超额完成省下达任务。

　　市本级经济发展全面提速，多项主要指标高于全市平均增幅。本级规模以上工业总产值完成650亿元、占全市规模以上工业总产值的比重达到54.2%，实现利润16亿元、比上年增长4.3倍；一般预算全口径财政收入完成31.2亿元、增长30.3%，地方级财政收入完成14.1亿元、增长33.1%，自2006年以来增幅首次超过全市平均水平；固定资产投资完成178亿元，增长45.9%，高于全市平均增幅9.2个百分点。

　　项目、招商和创新成效显著。实施3000万元以上项目806项、亿元以上项目190项，新上项目比重达到80.9%。通钢技术升级改造、祥元镁合金、安柏生物可得然胶和科泰新型材料产业园、弘美工业园、丰生医药园等18个10亿元以上项目快速推进，生物疫苗、白炭黑、医药包装新材料等一批亿元以上项目竣工投产。招商引资到位资金600亿元，实际利用外资2.1亿美元，引进亿元以上项目320项。凯丽国际工业博览城、丰汇润湾湾川生态新城两个“百亿元级”项目签约落地，四环制药、步长制药、神威制药、威高集团等国内百强医药企业和韩国浦项、美国辉瑞两户世界500强企业落户我市，四环制药当年落户、当年缴税3.2亿元。通化医药高新区与经济开发区整合获得省政府批准，国家级高新区已上报国务院审批。东昌、二道江、辉南开发区晋升省级开发区通过省政府核准验收。53个项目列入国家和省科技发展计划，国家创新药物孵化（通化）基地建设项目启动实施，新增国家高新技术企业5户。中国人参基因组图谱制作如期完成，中国北方药用物种基因组学转化研究中心正式成立，紫鑫、一洋等16户企业列入全省人参药食同源试点，长白山人参野生资源恢复工程全面启动，全省振兴人参产业现场会在我市召开。

　　工业经济增势强劲。规模以上工业增加值完成308亿元，增长28.4%；实现利润50亿元，增长73.6%，增速列全省首位，创历史最好水平。医药、冶金、食品三大支柱产业实现产值488亿元、255亿元、166亿元，分别增长30%、20%和56.7%，机械制造、建材、化工、能源等产业均实现较快发展。工业“五个一批”工程扎实推进，固定资产投资增长32%。开发新产品115种，新产品产值突破百亿元大关。新口径规模以上工业企业发展到450户，缴税超亿元企业由5户增加到9户。外贸进出口总额增长31%。新创中国驰名商标和“中华老字号”品牌各3件、国家地理标志保护产品2种。修正被认定为国家技术创新示范企业，万通获国家企业管理现代化创新成果奖，东宝、益盛两户企业的科技攻关成果获省科技进步一等奖。出台了加快保健品和金属镁产业发展指导意见。我市列入国家老工业基地试点城市，二道江区列入全国第三批资源枯竭城市转型试点。

　　农业农村经济快速发展。粮食产量突破39亿斤，创历史新高。实施千万元以上产业化项目20项，农产品加工量达到212万吨。新增绿色有机无公害农产品标识18个。柳河县被评为国家级水稻标准化生产示范县。建成标准化牧业小区226个，畜牧业产值增长22.4%。第二个“十年绿化美化吉林大地”活动全面启动，植树造林12.9万亩，实现连续57年无重大森林火灾。集体林权配套改革基本完成。实施新农村建设项目1643项，“百村示范、全面提升”工程和“清洁文明提升工程”取得新成效。67项乡村公路连通工程全面完成。实施了江河整治、水库除险加固、灌区节水改造等52项重点水利工程，412项水毁工程如期修复。提前一年完成302项水毁交通设施重建。化解农村义务教育剩余债务6060万元，农村专业合作社发展到1365个。农业信贷规模、土地流转规模和农业保险覆盖面进一步扩大，水文、气象服务功能得到提升。县域经济竞相发展，柳河县、梅河口市和辉南县进入全省县域综合发展指数排序前10位。

　　基础设施和城市建设实现新突破。编制完成特色城镇化发展规划，完善“两轴、两区、一带”和“一组两辅四组团”规划布局。城市总体规划通过省政府批准。完成新一轮土地利用总体规划修编，向国家和省争取到4857公顷新增建设用地指标。通丹、梅沈、营松高速公路通化段建成通车，通长、通集、通靖高速公路前期工作扎实推进，88项国省干线公路养护及建设工程按期完成。通化机场开工建设。沈阳桃仙机场通化候机楼投入运营。新建扩建66千伏变电站17座、线路19条。市区棚户区及危旧房改造拆迁83.6万平方米，开工100万平方米。“暖房子”工程完成改造300万平方米，是省下达计划的5倍；同步改造陈旧供热管网200公里，撤并改造小锅炉房56座；配套完成旧住宅小区环境综合整治50万平方米。平湖三期工程竣工，新增景观水面100万平方米。佐安河口至二道江大桥堤路改造工程全面完成，滨江东路北段全线贯通，主街路和50条巷路升级改造、绿化美化亮化等工程按时完成。解决了董窑沟等3个区域1万人全天用水问题，新增煤气用户4000户。实施饮用水源保护、医疗废物处理、烟气脱硫等环保工程，市区空气环境质量优良天数达到330天，比上年增加15天。加强城市环境综合整治，城市品位明显提升。

　　服务业发展步伐加快。通丹陆港开工，长白山医药物流中心、人参交易市场等项目全面推进。金融机构各项贷款余额达到399.4亿元，比上年增加56.7亿元。招商银行设通化分行，吉林省信用担保投资公司通化分公司正式组建，10亿元城建投资债券获准发行。益盛药业成功上市。英额布康乐谷等24个旅游项目启动实施，国内最大的火山溶洞—云霞洞完成一期开发并晋升为3A级景区，民俗特色旅游示范村建设完成年度工作目标，太王镇、金川镇被评为中国优秀旅游名镇。成功举办了中国通化松花砚（石）文化节系列活动，东昌区包装印刷产业园、万合影像婚庆基地等一批文化产业项目加快建设，关东文化产品交易市场被确定为吉林省文化产业示范园区。

　　民生进一步改善。城镇居民人均可支配收入达到18875元、增长13%，农民人均纯收入达到7950元、增长21%。制定实施民生工作计划，百件实事全面完成。城镇新增就业5.8万人，农村劳动力转移就业35.4万人。养老、失业、医疗、工伤、生育保险新增参保6.1万人。新型农村合作医疗参合率达到在乡农业人口的99.8%。新型农村社会养老保险试点参保率平均达到80%，高于省下达年度目标10个百分点。柳河县、通化县纳入新型农村和城镇居民社会养老保险试点。医疗、工伤、生育保险实行市级统筹，五七家属工参加基本养老保险工作在全市铺开。企业退休人员月人均养老金增加151元，城市低保月标准由214元提高到260元，农村低保年标准由1200元提高到1410元，城镇居民基本医疗保险和新农合人均补助标准提高至200元。“三帮扶”工作扎实推进。救助城乡困难群众16万人次。建设城市廉租房6200套，改造农村危旧房7000多户。市区发放住房租赁补贴20491户，无籍房登记确权2000户。建成农村饮水安全工程525处，解决了17.7万农村居民和1.9万农村师生饮水安全问题。启动新一轮“菜篮子”工程，新增蔬菜生产面积1.4万亩，棚膜蔬菜产量达到21万吨。“万村千乡市场工程”和“新网工程”深入实施。积极稳控市场物价，建立社会救助标准与物价上涨联动机制，保持了生活必需品价格基本稳定。

　　各项社会事业全面进步。义务教育均衡发展进程加快，校舍安全工程基本完成。基础教育有效加强，高考取得近20年最好成绩。职业教育校企合作办学模式改革成果显著，职教园区实训楼和特殊教育学校改扩建工程完工。市青少年宫正式启用，中小学综合实践基地建成开放。医药卫生体制改革和基本公共卫生服务均等化工作扎实推进，基本药物制度实施取得初步成效，各类传染病得到有效控制。引进3所特色专科医院，完成88所乡镇卫生院基础设施建设，社区卫生服务工作走在全省前列。市医院扩建、市中医医院和精神病院新建工程按期完成，梅河口市中心医院晋升为全省县级首家三级甲等综合医院。申报第三批省级非物质文化遗产项目实现新突破，自安山城抢救工程取得积极进展。集博物馆、科技馆、规划馆、美术馆和群众艺术馆为一体的市科技文化活动中心开工建设。市歌舞剧院和东方红剧场完成资源整合。在全省率先实现乡镇综合文化站全覆盖。广播电视户户通工程深入拓展。电视剧《远去的飞鹰》在国内多家电视台播出。竞技体育获省级以上金牌59枚。完成了第六次全国人口普查。

　　民主法制建设得到加强。自觉接受市人大及其常委会的法律监督、工作监督和市政协民主监督，办理人大代表建议107件、政协提案155件。“六五”普法启动实施。坚持依法行政、从严治政，廉政建设取得新成绩。推行全程、预约、咨询、承办、快捷“五项服务”，深化政行风民主评议和纠风专项治理，软环境进一步改善。探索社会管理创新，建立人民调解、行政调解与司法调解联动的调解格局，维稳工作机制日趋完善。调解矛盾纠纷9400余件，“N+1”矛盾纠纷排查调处经验在全国推广。高度重视社会舆情和群众诉求，着力解决信访问题，信访、进京上省非正常访数量“双下降”。市长公开电话受理群众来电5.3万件，办复率99%。精心组织安全生产大排查、大整治、大培训、大练兵、大宣传系列活动，集中开展“清剿火患”战役和交通安全整治等专项行动，事故起数和死亡控制指标实现历史最好水平。加强食品药品安全监管，主办了全国食品药品系统科学监管通化交流峰会。深入开展平安通化建设，扎实推进打黑除恶、命案侦破等专项行动和“打四黑除四害”、打击“地沟油”违法犯罪专项整治，社会治安集中整治成效显著。加大“天网工程”建设力度，城区重点区域、主要街道实现视频监控全覆盖。

　　在即将过去的一年里，通化被联合国环境规划署评为中国区环境规划优秀示范城市，获得中国最具国际影响力旅游城市、中国十佳品牌城市、中国低碳生态先进城市、全国双拥模范城“七连冠”、全国地名公共服务示范市、全国交通运输文明单位、全国人口计生工作先进单位、全国青少年普法教育先进单位等荣誉。

　　这些成绩的取得，是市委正确领导的结果，是市人大、市政协监督支持的结果，是全市人民团结奋斗的结果，是社会各界共同参与的结果。在此，我代表市政府，向辛勤工作在各行各业的全市人民，向给予政府工作大力支持的人大代表和政协委员，向各民主党派、工商联、无党派人士和人民团体，向驻通国省属企事业单位、驻通部队和武警官兵，向所有关心支持通化发展的同志们、朋友们，表示衷心的感谢！

　　在肯定成绩的同时，我们也清醒地看到，我市经济社会发展还存在一些问题，主要是总量还不大，自主创新能力还不强，转变发展方式任务繁重；中心城市规模小，尚未形成较强的辐射带动功能；就业和社会保障压力较大，部分低收入群众生活还很困难，改善民生需要付出艰苦努力；社会管理和公共服务水平有待提高，一些影响和谐稳定的问题需要进一步解决；软环境建设还有差距，还存在部分公职人员责任意识和服务意识不强、工作作风不实等问题。这些问题，我们要采取有力措施，认真加以解决。

　　二、2012年目标任务

　　2012年，市政府要坚持以科学发展观为指导，认真贯彻落实党的十七届六中全会、省委九届十二次全会和市第六次党代会精神，以科学发展为主题，以加快转变经济发展方式为主线，以改革开放为动力，以保障和改善民生为根本，大力推进具有通化特色的工业化、城镇化、农业现代化“三化”统筹，强力实施投资拉动、项目带动、创新驱动“三动”战略和富民工程，全面加强经济建设、政治建设、文化建设、社会建设和生态文明建设，坚定不移地抓项目、兴产业、惠民生、促发展，加快建设东北东部地区中心城市、吉林省东南部新型工业基地、中国北方宜居山水城市，不断迈出科学发展、进位赶超、振兴崛起的新步伐。

　　主要预期目标：地区生产总值增长20%，财政收入与经济同步增长，固定资产投资增长30%，社会消费品零售总额增长17%以上，城镇居民人均可支配收入和农民人均纯收入分别增长12%，城镇登记失业率控制在4.6%以内，单位地区生产总值能耗和主要污染物减排完成省下达指标。在工作安排上，要紧紧把握国家政策导向，积极应对外部环境变化，自我加压，强化措施，力争主要发展指标和收入指标实现更大幅度增长。

　　完成新一年目标任务，推动通化平稳较快发展，必须坚持把发展作为第一要务，以创新发展为引领，以和谐发展为保障，在发展中争创优势、实现跨越；坚持增量调结构、创新促升级、发展保民生，把经济发展方式转换到内生增长、创新驱动的轨道，把社会管理方式转换到以人为本、服务为先的轨道；坚持一切为了人民、一切依靠人民，切实提升群众的满意度和幸福感。

　　（一）加大项目建设和招商引资力度。新建续建3000万元以上项目820项，其中亿元以上项目200项、10亿元以上项目25项。加快凯丽国际工业博览城、丰汇润湾湾川生态新城两个“百亿元级”项目运作，推进东宝人胰岛素注射剂三期工程、四环医药工业园、步长北方基地、神威中药材种植基地及北方生产基地、威高保健品生产基地、双龙硅化工产业园、华能热电等项目实施，修正斯达舒产业化扩能改造、通钢新2号高炉、凤形耐磨材料、安柏生物可得然胶等项目建成投产。抓住国家编制新一轮老工业基地调整改造规划等机遇，精心做好项目谋划，积极争取国家和省里的项目资金支持。继续实行重大项目推进责任制，加强项目建设服务、监督管理和跟踪审计，做好土地、林地征用、环评和被征地农民补偿安置等工作。创新招商方式，拓宽引资渠道，落实招商引资目标责任制，瞄准国内外500强、央企、重点上市公司、知名品牌公司、大型民企，着力引进一批战略性大项目。引进亿元以上项目200项，引进资金660亿元，实际利用外资2.3亿美元。

　　（二）强力推动产业结构优化升级。深入实施八大工业产业跃升计划，支持现有企业扩能改造、盘活存量，加速装备、技术和产品全面升级，确保工业提速增效。规模以上工业总产值达到1500亿元，增长25%。突出发展现代中药和生物制药，引进一批知名医药企业，开发一批重大创新药物，进一步提高医药产业集聚度。通化医药高新区开工、投产超亿元项目各达到10个以上，落地千万元以上项目50个，争取晋升为国家级高新区。推动冶金产业园区与通钢对接，建设“五鸭铁”兴钢新区、浦项钢材加工中心项目，做大钢铁深加工产业集群，提高钢材就地转化率和制品化率。建成5万吨镁合金项目，拉长镁合金上下游产业链条。实施“一带、双百、三区”培育计划，尽快把保健品产业培育成为新的优势产业。支持通葡股份等葡萄酒企业健康发展，振兴通化葡萄酒品牌。建成通天葡萄酒科技园区。大力发展新材料、新能源、节能环保、先进装备制造等战略性新兴产业，加快发展石油机械等优势产业。加强资源勘探和规范管理，推进矿业产业发展。抓好三年滚动技术改造工程，完成技改投资180亿元。实施品牌战略和标准化战略，新创一批驰名商标、名牌产品和地理标志保护品种。实施龙•溪谷滑雪小镇、白鸡峰旅游经济区等45个重点旅游项目，开展高端媒体营销，打造一批精品旅游线路。力争开通松江河至集安铁路旅游客运专线。加快江南新区、通化县、梅河口物流园区建设，推进长白山医药物流、三源浦空港经济区等项目实施。加快国家级苗木交易市场和粮油、农资等区域批发市场建设。加强房地产市场监测和市场行为监管，促进房地产业健康发展。大力发展市区经济，着力抓工业、抓产业、抓税源项目、抓后劲培育，做大总量、做强实力，使市区成为全市科学发展的领头羊、加快振兴的排头兵。

　　（三）抓好县域突破和农业农村发展。推进县域开发区提档升级，7个县（市、区）开发区新上10个超5亿元的工业项目，柳河、通化县经济开发区争取晋升为省级开发区，县域工业投资占比都要超过50%。推进县城和中心镇发展，增强综合承载功能，吸纳农村人口集聚。开展城乡双向一体化试点，推动曙光、朝阳、三源浦、大安、五道江、金厂、清河等节点城镇和特色镇、中心镇加快发展。实施新农村建设项目1540项，继续抓好“百村示范、全面提升”工程、“清洁文明提升工程”和民俗特色旅游示范村建设，113个省级示范村全部达标。推进现代农作物种业发展和粮食增产增效示范区建设，稳产高产良田达到270万亩以上。抓好20个千万元以上农业产业化重点项目，启动建设吉煤集团特色农牧业产业基地项目。新建130个标准化牧业小区，畜牧业产值增长20%。大力发展高效特产业，特产业产值增长20%以上。推进人参产业振兴，抓好长白山人参贸易区和康美、东宝人参产业园建设，积极开发药食同源新产品，提高人参种植规模化、质量标准化、加工精深化、产业集约化水平。落实林业产业倍增计划，提高林业经济效益。加大“菜篮子”工程实施力度，规划建设20个棚膜蔬菜生产基地，新增棚膜菜田4000亩，增加地产菜供应能力。

　　（四）全面加快基础设施建设。重点抓好十项工程：一是公路工程。加快通长、通集、通靖高速公路前期工作，争取尽早开工。力争开工建设官道岭互通立交桥，开通市区绕越线出口。实施普通公路养护工程8项、建设工程203项。二是铁路工程。通丹铁路建成通车，积极推进四平至通化至松江河快速铁路，筹划沈吉复线梅河口至辉南段及通集快速铁路。三是机场工程。完成通化机场建设，力争实现通航。四是陆港工程。通丹陆港年内封关运行。五是电力工程。滚动实施“十二五”电网规划，加快大中型电网和农网改造升级，新建220千伏线路1条、66千伏线路10条、变电站11座。六是水利工程。实施江河整治、水库除险加固等65项水利工程。七是环保工程。开工垃圾焚烧发电工程，建成二道江污水处理厂。八是水源工程。完成市区备用水源前期工作。九是土地开发整理工程。申报复垦耕地25万亩，开发整理土地120万亩。十是信息产业工程。推进“两化融合”示范工程，加快“数字通化”和无线城市信息平台建设。

　　（五）大力提升城市规划、建设和管理水平。编制完成城市控制性详规和5个专项规划。市区城建计划投资45亿元。加快棚户区及国有工矿棚户区改造，拆迁71.2万平方米，开工建筑面积95万平方米，重点改造中心城区，推动改造向周边延伸。继续高质量实施“暖房子”工程，完成改造100万平方米，改造陈旧管网100公里，综合整治旧住宅小区40万平方米。启动集中供热四期工程，提高集中供热覆盖面。完成盛和药业至修正大桥段堤路改造工程，全线贯通滨江东路。新建续建江南新区11条路网，改造周山巷路50条。对玉皇山桥、红旗桥、江南大桥进行景观改造，完成西出口和北出口改造。抓好绿化亮化硬化，提升绿化亮化档次，消除硬化死角。扩大巷路亮化覆盖，亮化黑楼道200栋。解决高区及周边1.8万户居民用水、5000户居民用气问题。继续推进国家卫生城市、园林城市、环保模范城市和全国文明城市创建工作，深入开展城市环境综合整治，强化商贸市场、街路巷路、居民小区和城乡结合部卫生保洁，有效治理“野广告”、焚烧冥纸、宠物造脏等违规行为。进一步抓好物业管理。实施城市畅通工程，抓好城市交通管理，完善交通设施和交通管控，逐步解决丁字路、断头路和停车难等问题。

　　（六）促进文化繁荣发展。制定文化事业和文化产业发展规划，在全市开展“文化年”活动。实施四项文化工程：一是文化强基惠民工程。推进图书馆、档案馆、博物馆、群众艺术馆、文化馆、科技馆新建扩建和改造，完善乡镇综合文化站服务功能，争取实现行政村文化大院和农家书屋全覆盖。二是地域历史文化资源建设工程。编制重要遗址保护规划，加强高句丽文化及世界文化遗产地的保护、开发和利用，推进自安山城、罗通山城遗址保护。成立非物质文化遗产研究院，做好国家第四批非物质文化遗产项目申报工作。深入挖掘自然风光、历史遗存、红色经典、民族民俗、工艺美术等文化资源内涵，促进文化与旅游、商贸、物流等产业融合发展。三是名人名师名家名品培育工程。启动文化人才培养计划，培养一批拔尖人才、专门人才和复合型人才。配齐配强基层文化管理干部。建立文化奖评机制，选树满族剪纸、艺发刻纸、松花石雕刻技艺和戏剧创作等领域的文化领军人物，加强代表性传承人命名和保护。传承开发、整合提升中医药、松花砚（石）、人参、葡萄酒和大泉源白酒的文化创意元素，努力打造“国药”、“国砚”、“国参”、“国酒”等文化品牌。围绕地域文化特色和重点产业，创作一批优秀文艺作品。推出舞台剧目《人参仙子》，筹拍《参王》等电视剧，发行全国首部《文化大院—文艺演唱作品集》。推进国有文艺院团转企改制，组建歌舞演艺集团公司。四是文化产业发展提速工程。推动文化与地域特点和产业特色深度融合，加大中医药文化的挖掘、传承、创新和发展力度，为中药产业发展提供文化支撑。筹建华夏中医科学院通化分院，申报一批中医药科研项目。实施关东文化产品交易市场二期工程，扶持松花砚产业特色化、品牌化、规模化发展。办好中国通化松花砚（石）文化节系列活动。加快包装印刷、满族民俗、影视婚庆、休闲养生等文化产业园区建设，发展英语辅导报等文化出版产业，筹建文化创意产业园和文化主题公园。

　　（七）进一步深化改革、扩大开放。积极推进企业兼并重组和产业链整合，稳步推行厂办大集体企业改革。抓好企业一套表改革工作。落实民营经济腾飞计划，扶持小型微型企业发展。深化农村综合改革，健全土地承包经营权流转市场，发展农民合作组织，完善农村现代流通体系。深化户籍制度改革，逐步满足符合条件的农村人口进城落户需求。壮大融资平台，设立政府引导基金，改善中小企业融资服务。加快旅游体制改革，推动旅游资源一体化。加强与东北东部城市合作交流，密切对接长吉图，积极融入环渤海，加快建设通丹经济带。推进鸭绿江界河大桥和集安铁路大桥规划建设，提高过货通关能力。加强出口基地建设，把更多的地产品推向国际市场，实现外贸进出口总额7.1亿美元。

　　（八）加强节能环保和生态建设。加快建设资源节约型、环境友好型城市，倡导绿色、低碳生产方式和消费模式。抓好节能降耗和减排治污，组织企业开展对标达标。有序开发特色资源，因地制宜发展农村新能源，集约节约利用土地。推行清洁生产，抓好循环经济试验区建设，提高资源综合利用效益。加强环境保护监督管理，推进城市污水处理厂达标排放。开展农村环境连片整治，提高各类污染防治水平。全面开展哈泥河流域集中整治，强化饮用水源的保护和监管，实现水质状况进一步改善。搞好江河及公路、铁路沿线环境治理，打造沿河沿线生态带、旅游带、城镇带。实施第二个“十年绿化美化吉林大地”规划，完善生态补偿机制和生态保护区扶持机制，保护森林、湿地资源。植树造林10万亩，标准化绿化村屯300个。启动市区周山治理工程，用两至三年时间完成周山棚户区拆迁改造。争取东昌区纳入长白山林区生态保护与经济转型试点。

　　（九）协调发展社会各项事业。完成科技创新“310”工程，申报国家和省科技专项60项，争取列入全省创新型城市试点、国家知识产权示范城市。筹建科技创新中心、科技企业孵化器、省级重点实验室，探索建立省级健康医药产学研创新联盟，争创国家医药物联网示范基地。强化素质教育，推进义务教育均衡发展，支持普通高中办出质量和特色。发展学前教育，加强幼儿园建设。改革职业教育办学模式，推动校企合作办学。支持通化师院加快发展。抓好人才引进和培养，造就更多的高素质劳动者和创新型、创业型人才。深入推进医药卫生体制改革，整合医疗卫生资源，完善医疗服务体系，巩固基本公共卫生服务和基本药物制度，加强重大传染病防控，增强医疗卫生服务保障能力。稳定适度低生育水平，降低出生缺陷发生率。开展全民健身活动，争取第十二届全国冬运会取得好成绩，积极备战第十七届省运会。保障妇女儿童权益，发展老龄、残疾人事业和慈善事业。依法管理宗教事务，增进民族团结。做好人防、地方志等工作。加强双拥共建和全民国防教育，开展新一轮全国双拥模范城创建活动。市区重点实施十个公益事业项目：一是建成市科技文化活动中心；二是建成城市规划展览馆；三是完成市一中、通化师院分院等校舍改造工程，推进十三中迁址新建；四是新建市中心医院外科大楼、市儿童医院；五是完成西山体育场、通钢游泳馆改造，建成西山和中东2个全民健身中心；六是建设人口与家庭综合服务中心；七是规划建设社区文化活动中心；八是建成残疾人康复中心；九是建设应急指挥中心和救灾物资储备库；十是新建蔬菜综合批发市场，建成蔬菜储备库。

　　（十）全力改善民生、创新社会管理。制定实施第三个民生工作计划，在富民增收、创业就业、社会保障、社会救助、扶贫济困等方面集中力量办一批实事。加大创业培训补贴和小额担保贷款力度，新增个体工商业户8500户。农村劳动力转移就业30万人。城镇新增就业5.5万人，确保“零就业家庭”至少一人实现就业。建立职工工资集体协商机制、正常增长机制、支付保障机制，完善公务员工资制度和事业单位分配制度。继续提高基本养老金、失业保险金、城乡低保、城镇居民基本医疗保险、新型农村合作医疗补助标准和低保对象大病医疗救助标准，对低保对象五种重大疾病治疗给予全额救助。抓好城镇居民和新型农村养老保险试点。市区建成廉租房2万平方米，为1.7万户低收入困难家庭发放住房租赁补贴，完成3000户无籍房屋确权工作。改造农村危旧房5000户。建成农村饮水安全工程310处，解决14.5万农村人口饮水安全问题。整合基层社会管理服务资源，加快构建源头治理、动态协调、应急处置互相联系和支持的社会管理体制机制。创建柔性服务、精细管理、常态治理、民主管理机制和信息系统，完善群众利益协调、诉求表达、矛盾调处和权益保障机制，认真做好信访工作。大力开展平安通化创建活动，完善社会治安防控体系，严厉打击各类犯罪。深入开展安全生产“五项”工作，以煤矿、消防、道路交通、建筑施工、森林防火、校园安全、校车安全等领域为重点，开展经常性的隐患排查和治理，遏制重特大事故发生。严格食品药品安全监管，坚持开展各类专项整治，让人民群众吃上放心食品、用上放心药品。建设平价商店，加强物价监管，稳定生活必需品市场价格。

　　三、加强政府自身建设

　　大兴紧密联系实际之风、务实重行之风、改革创新之风、艰苦奋斗之风、密切联系群众之风，切实转变政府职能，推进政府管理创新，提高政府公信力、执行力和推动力，努力建设法治政府、服务政府。

　　全面推进依法行政。自觉接受市人大的法律监督和工作监督，主动接受市政协的民主监督和参政意见，认真做好人大代表建议和政协委员提案办理工作。重视人民群众监督和新闻舆论监督，加强与公众直接沟通互动。落实行政执法责任制，严密执法程序，强化监督检查，做到严格执法、公正执法、理性执法、文明执法。制定便民措施，畅通行政复议渠道。整体推进“六五”普法。扩大法律援助覆盖面，维护社会公平正义。

　　坚持科学民主决策。健全重大行政决策规则，把专家论证、决策咨询、风险评估、合法性审查和集体讨论决定作为重大决策的必经程序。广泛听取群众意见，深入了解社会反映，使各项决策更加符合实际、经得起检验。健全重大决策部署实施评价、反馈纠偏和责任追究制度，及时发现并解决执行过程中的问题。推动政务公开向基层延伸，健全规范信息公开和新闻发布制度，保障人民群众的知情权、参与权和监督权。

　　着力提高行政效能。加强各级政务大厅行政审批、公共服务、资源交易、效能监察四个平台建设，加快推进网上审批试点。实行重点项目行政审批“直通车”，推行一次告知、一站办理、一车踏勘、一单收费，提高行政审批即办比率。建立健全重点工作、重大项目分工包保机制、推进落实机制和协同协调机制，深入推进“查找改”，使决策、执行、监督有效链接。整治和建设软环境，推行绩效管理，严格行政问责。

　　积极改进工作作风。解放思想，振奋精神，创造性地开展工作。把心思和精力用在落实上，敢担当，敢面对，不回避矛盾，迎着困难上。精简会议和文件，改进会风文风。牢固树立服务思想，深入企业、深入基层、深入群众，使问题在一线发现，矛盾在一线解决，工作在一线落实，能力在一线提升，成效在一线体现。

　　认真抓好廉政建设。严格落实廉政建设责任制，注重工程招投标、土地招拍挂、大额资金使用、国有资产处置、政府投资项目等重点领域源头控制。建设学习型机关和高素质公务员队伍，营造风清气正、勤勉敬业、干净干事的政务环境。

　　各位代表！通化正处在振兴发展的关键时期，形势催人奋进，使命无尚光荣。让我们在市委的正确领导下，在市人大、市政协的监督支持下，凝心聚力，只争朝夕，务实创新，奋发图强，全力推进通化科学发展、进位赶超、振兴崛起，为全市人民生活得更加幸福美好而努力奋斗。
